# Origin DNA Melting—An Essential Process with Divergent Mechanisms

**DOI:** 10.3390/genes8010026

**Published:** 2017-01-11

**Authors:** Matthew P. Martinez, John M. Jones, Irina Bruck, Daniel L. Kaplan

**Affiliations:** Department of Biomedical Sciences, Florida State University College of Medicine, 1115 W. Call St., Tallahassee, FL 32306, USA; mm14d@my.fsu.edu (M.P.M.); jmj13b@my.fsu.edu (J.M.J.); irina.bruck@med.fsu.edu (I.B.)

**Keywords:** DNA helicase, DNA replication, initiation, protein-DNA interaction, DnaA, Large T antigen, E1 helicase, Mcm2–7, melting

## Abstract

Origin DNA melting is an essential process in the various domains of life. The replication fork helicase unwinds DNA ahead of the replication fork, providing single-stranded DNA templates for the replicative polymerases. The replication fork helicase is a ring shaped-assembly that unwinds DNA by a steric exclusion mechanism in most DNA replication systems. While one strand of DNA passes through the central channel of the helicase ring, the second DNA strand is excluded from the central channel. Thus, the origin, or initiation site for DNA replication, must melt during the initiation of DNA replication to allow for the helicase to surround a single-DNA strand. While this process is largely understood for bacteria and eukaryotic viruses, less is known about how origin DNA is melted at eukaryotic cellular origins. This review describes the current state of knowledge of how genomic DNA is melted at a replication origin in bacteria and eukaryotes. We propose that although the process of origin melting is essential for the various domains of life, the mechanism for origin melting may be quite different among the different DNA replication initiation systems.

## 1. Review of Bacterial Replication Initiator DnaA

Like every other organism, bacteria must replicate their DNA in order to produce viable offspring. However, bacteria cannot infinitely replicate, meaning there must be a tight regulation of this process. The fact that replication does not just start and pause indicates that there is a lot of regulation on the initiation of chromosome replication. DnaA, the key initiator protein among almost all bacteria, is a highly conserved protein and is the driver of the system in which DNA replication initiation is regulated. This protein has been studied extensively and understood through the *Escherichia coli* model.

## 2. DnaA-Orisome Structure

**Figure 1:**
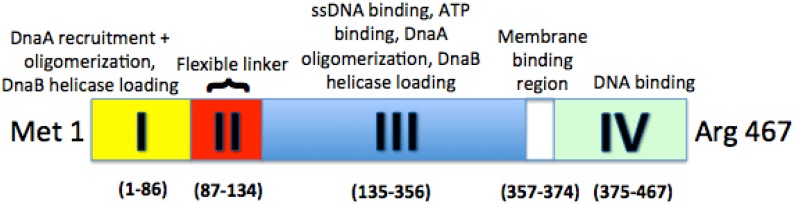
A schematic map of the four domains of DnaA. ssDNA: single-stranded DNA.

**Figure 2:**
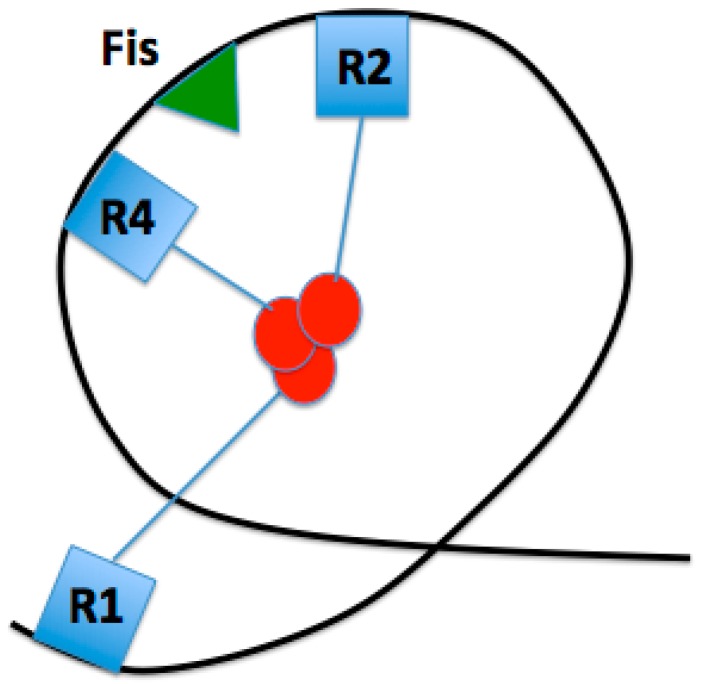
Proposed loop conformation of inactive oriC, constrained by DnaA bound to high-affinity sites R1, R2, and R4 via domain I N-terminus interactions. This conformation is facilitated by Fis.

DnaA is a key protein in the initiation of bacterial replication (Figure 1). Bound to high- and low-affinity sites at the initiation sequence, *oriC*, DnaA is a highly conserved protein among all bacteria that comprises the DNA-protein complex termed the orisome, which triggers the initiation of chromosome replication. OriC DNA is not bare throughout the cell cycle, but instead has bound DnaA to three high-affinity sites (left to right: R1, R2, R4). These three DnaA sites, along with oriC bending protein Fis, set a nucleosome-like conformation in the origin that has been suggested to prevent replication initiation (Figure 2) [1]. Fis is not necessary for viability, however, the lack of Fis binding results in asynchronous replication in rapidly growing cells. This is due to the binding of DnaA to low affinity sites at a lower concentration than what is normally required, since there is no Fis protein to inhibit DnaA binding [2]. Additionally, this conformation keeps the DNA double-stranded until the appropriate replication-promoting proteins bind and separate the two strands. The review by Leonard and Grimwade [1] discusses that these replication-promoting proteins include additional DnaA and another DNA bending protein, Integration Host Factor (IHF). Upon accumulation of a sufficient level of DnaA-ATP, the active form of DnaA, Fis will be displaced and IHF will bind, along with DnaA, to low affinity sites between R1 and R2, and R2 and R4 [3]. IHF has been shown to be nonessential for the assembly of a functional orisome, however, this loss of IHF results in perturbed replication initiation [1]. The viability of cells lacking IHF binding is most likely due to the flexibility of the DNA between R1 and R5M.

Although the exact mechanism of the displacement of this initiator inhibition is unclear, a recent study has shown that ATP-bound DnaA, as opposed to ADP-bound DnaA, experiences a conformational change within domains I–III that enhances its ability to bind to low affinity sites within oriC as well as cooperatively bind to already bound DnaA molecules [4]. Once a threshold concentration of DnaA-ATP is achieved in the cell, Fis can successfully be displaced and the inhibitory complex can progress to an active one. DNase I footprinting studies have suggested that DNA wraps around the DnaA oligomer once bound [5]. As illustrated in Figure 1, the function of each domain has been determined via reverse genetics: DnaA recruitment (I), DNA binding (IV), oligomerization (I, III), ATP binding (III), and helicase loading (I, III) [1]. Between domains III and IV is an amphipathic region that is involved in binding to the inner membrane of the cell [6]. Additionally, domain II serves as a flexible linker, aligning domain I with domains III + IV [4]. 

## 3. DNA Conformation

**Figure 3:**
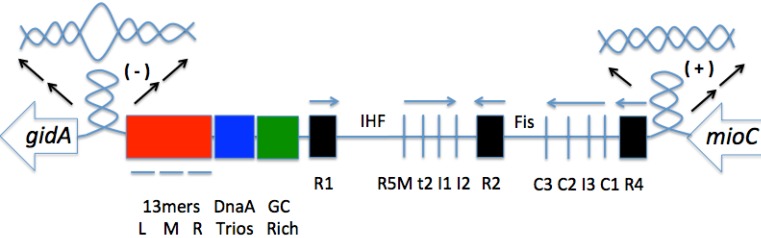
The origin of replication in *Escherichia coli*, oriC. This 245-bp sequence consists of the 13-mer DNA unwinding element (red), DnaA-trio motifs (blue), and binding sites for DnaA, Integration Host Factor (IHF), and Fis. Additionally, flanking genes *gidA* and *mioC* are shown. The arrows represent the transcription direction of the flanking genes (large, hollow arrows) and directionality of DnaA filament formation (small arrows above DnaA boxes). The black arrows help visualize each type of supercoiling, shown above the oriC*.*

The oriC DNA contains multiple sites of DnaA binding in which specific binding is required for duplex unwinding (Figure 3). Between the three high affinity sites mentioned in the above paragraph are low affinity DnaA sites (R5M, τ2, I1, and I2, respectively, between R1 and R2; C3, C2, I3, and C1, respectively, between R2 and R4) [3], which become DnaA bound just before origin melting. The left half of oriC (R1–I2) and right half of oriC (R2–R4) have opposite orientations, with both oriented inward of oriC (towards each other) [3]. Kaur et al. demonstrated that the loss of any two high affinity sites resulted in the loss of oriC function, while the loss of any single high affinity site resulted in a functional oriC with perturbed initiation timing, with an R4 mutation being the most significant [3]. The loss of R2 showed the least significant impact, implying that R2 may be a redundant site or may stabilize the oligomers from R1 and R4. The loss of any single high affinity site rendered the cell dependent on both Fis and IHF binding for a functional oriC. When either R1 or R4 was deleted, R2 was shown to be capable of nucleating a DnaA oligomer, although a higher concentration of DnaA was required. Less DnaA was detected in the right half of oriC in R4 mutants, supporting the importance of R4 [2]. Additionally, it was shown that *E. coli* mutants with a deletion in the entire right half or oriC (R2–R4) are still viable under slow growth conditions. However, with sensitivity to rich media and other rapid growth conditions, it is possible that the right half of oriC has evolved to support multi-forked replication [7]. With these data on alternative methods for pre-RC formation, the minimum requirements for origin melting can be further investigated and understood with greater complexity.

The DNA unwinding element is an AT-rich region towards the left of oriC that has less helical stability than the rest of oriC DNA. DNA unwinding element (DUE) consists of three regions (L, M, R) 13-mer repeats [1]. The DUE is the first piece of DNA to unwind in replication initiation [8], with evidence supporting initiation of melting beginning with the L-region [9]. Kowalski and Eddy have demonstrated that by deleting the l-13mer and replacing it with a dissimilar sequence, its helical instability, rather than its specific sequence, is essential for origin function and duplex unwinding. Meanwhile, the sequence of the r-13mer is the most evolutionarily conserved of the three segments, suggesting a role for the r-13mer in specific protein recognition [10].

An increase in net negative supercoiling (a general undertwist in the DNA has been shown in more efficient *E. coli* initiation, indicating that this chromosomal topology is preferred for replication initiation [11]. The flanking gene *gidA* introduces negative supercoiling to the left of the DUE, which helps further destabilize the already less thermodynamically stable AT-rich DUE [11]. Supporting this, maximal *gidA* transcription occurs before initiation. Additionally, Magnan and Bates discuss in their review the importance of positive supercoiling in regulating oriC transcription [11]. The positive supercoiling to the right of DUE is regulated by the flanking gene *mioC*, with maximal transcription immediately after initiation. While *gidA* and *mioC* are both dispensable, it is possible that they help drive initiation under suboptimal conditions [11]. Kaur et al. tested for the conformation of oriC pre-melting, and developed a model in which oriC forms a constrained loop by interactions of the N-termini of high affinity-bound DnaA, and this loop and repression of active low affinity sites is assisted by Fis binding (Figure 2) [3]. It is possible that this pre-initiation complex causes a reduction of negative supercoiling adjacent to the DUE, and further research is needed to support this.

Recent research has found a DnaA-trio, which consists of a repeating trinucleotide motif, beginning with 3′-GAT-5′, which lies between the AT-rich DUE and the GC rich region (which is adjacent to the DnaA boxes) (Figure 3) [12]. These newer findings will be discussed in greater detail later on. 

## 4. Initiator Mechanism

DnaA contains various AAA+ (ATPases Associated with various cellular Activities) motif sequences which provide a range of functions, including DnaA-DnaA binding [13] and DnaA-ssDNA (single-stranded DNA) binding [14]. DnaA bound to oriC high affinity DNA boxes, via its domain IV helix-turn-helix motif [4], nucleates by binding ATP-DnaA at adjacent low affinity sites. Interestingly, one method of regulation of this step is through a chromosome-membrane protein tether. Bound to an array of operator sequences on the chromosome up to 1 Mb away from oriC, this tether is proposed to inhibit DnaA binding to DNA by reducing the net negative supercoiling [15], although this mechanism is not quite yet understood. DnaA-ATP is required for effective binding to low affinity sites and DnaA oligomer formation [4], yet DnaA cannot always be bound to ATP. Examining the crystal structure of DnaA bound to ssDNA revealed four DnaA protomers per oligomer, forming a right hand spiral around a single strand of the duplex DNA [14].

The DnaA oligomer formation from R1 and R4 inwards towards R2 [3] is mediated by the Arg285 residue within domain III, which is oriented inward towards R2 for both the right and left half of oriC [13]. These Arg285 fingers stimulate subcomplex formation by binding the ATP nucleotide of the next DnaA monomer, eventually forming a DnaA oligomer. This study also found that the Arg285 finger of R1-box-bound DnaA is crucial for DUE unwinding and single-stranded DNA unwinding element (ssDUE) binding, where the same residue of R4-box-bound DnaA plays a necessary role in DnaB helicase loading.

**Figure 4:**
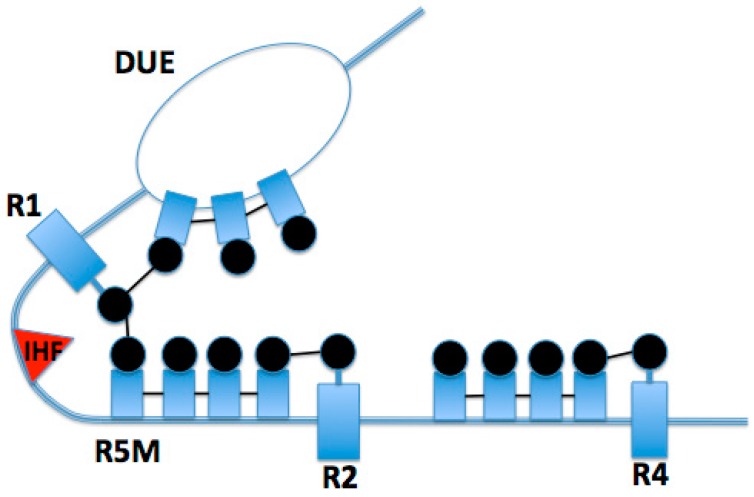
Active oriC conformation, showing interactions between R1 and R5M facilitated by IHF, and interactions between R1 DnaA and DNA unwinding element (DUE)-bound DnaA, which facilitates filament formation on ssDNA. The thicker blue line represents double-stranded oriC DNA, and the thin lines of the “bubble” represent the single-stranded DNA of the melted DUE.

The interaction between DnaA monomers facilitates a conformational change in the bound strand of DNA, stretching the contacted strand and disrupting the base pairs of the thermodynamically unstable DUE [14]. Once this region of oriC unwinds, origin melting is enhanced by binding of the DnaA box-bound DnaA filaments to the partially melted region of oriC DNA. DnaA forms a helical filament around the ssDNA, where each protomer binds three nucleotides via two pairs of helices, α 3/α 4 and α 5/α 6, which line the inner channel of this protein assembly [14]. Additionally, this conformation prevents reannealing of the two strands of DNA. Once this conformation is set, as visualized in Figure 4, DnaA stabilizes the partially melted origin by nucleating from the already bound DnaA, forming dynamic filaments on the ssDNA monomer by monomer in a 3′-5′ directionality [16]. 

While the details of this mechanism have been widely unknown, a recent study has identified a DnaA-trio motif within the ssDNA, which is recognized by the DnaA box-bound DnaA [12], facilitating filament formation on the ssDNA via the domain III Initiator Specific Motif (ISM) Initiator Specific Motif [14]. According to the study conducted by Richardson et al., the box-bound DnaA recognizes a 3′-GAT-5′ sequence, with some variability between the first and third nucleotide, but a highly conserved second adenine nucleotide [12]. At this point, DnaA will nucleate across the next few DnaA-trios and into the DUE. Upon filament formation and further duplex melting, DnaA will load DnaB via domain I and domain III interactions, initiating the formation of the prepriming complex (Figure 4, [17]).

## 5. Large T-Antigen and E1 Helicases

Mechanisms of origin melting can be derived from the structural analysis of the DNA tumor virus Simian virus 40 (SV40) and papillomavirus. Specifically, SV40 utilizes its Large T antigen (LTag) to initially separate and continually unwind double-stranded DNA (dsDNA) in host cells, and the papillomavirus enlists E1 to do the same. Due to eukaryotic similarities, such as homohexameric domains and beta hairpin loops, results derived from these models may be applicable to the understanding of eukaryotic melting processes. Unlike replication in eukaryotes, melting with initiators SV40 and E1 is performed through cooperation of only a handful of protein domains compared to the variety of protein complexes often necessary to facilitate eukaryotic DNA replication. This lack of complexity yet abundance of shared homology has allowed recent studying of SV40 and E1 to elucidate potential mechanisms for eukaryotic origin melting.

## 6. Structure of LTag and the Core Ori

**Figure 5:**
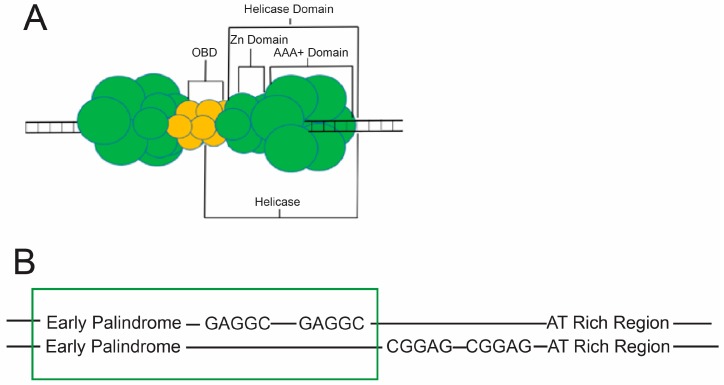
Structure of the Large T Antigen (LTag) and the Simian Virus 40 (SV40) Core Ori. (**A**) A cartoon model illustrating the double hexameric LTag complex and its relevant subdivisions. A single hexamer is noted to contain a portion of the origin binding domain (OBD) and a helicase domain, which itself includes a Zn and AAA+ domain; (**B**) Depiction of the Core Ori of SV40 viral double-stranded DNA (dsDNA) including the four GAGGC pentamers and the flanking AT-rich (AT) and early palindromic sequence (EP) regions. The box around two pentamers and the EP region indicates what portion of the core ori a single hexamer of LTag would occupy.

Melting of eukaryotic DNA is thought to require a variety of protein factors which work together to manipulate dsDNA, ultimately separating the two strands via mechanical force. Due to the complexity of the eukaryotic cellular machinery, researchers have turned to more simplistic models of initiation, such as the Large T antigen. LTag is a double hexameric protein complex produced by the SV40 virus which is solely responsible for melting of SV40 viral DNA origins, as well as helicase activity once replication forks have been established. Three distinguishable domains compartmentalize these actions, the first of which is known as the origin binding domain (OBD). The OBD of LTag has been shown to bind both dsDNA and ssDNA [18] much like the DNA binding domains of DnaA [12]. Many similarly structured DNA binding domains (DBDs) of eukaryotic and prokaryotic replication machinery bind ssDNA specifically, such as eukaryotic replication protein A (RPA), and bacterial *E. coli* single-stranded DNA-binding proteins (EcoSSB) [19,20,21]. The second and third domains are the Zn domains, and AAA+ domains, respectively [22]. The three domains can be found in Figure 5A. To initiate replication, these domains seek out designated binding sites on viral DNA along a segment known as the core origin of DNA.

**Figure 6:**
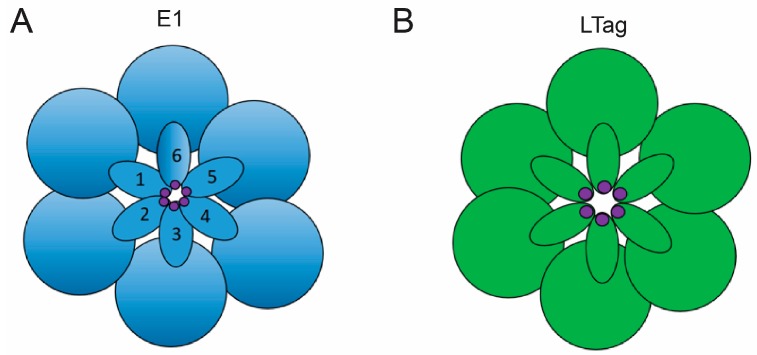
E1 vs. LTag Beta Hairpin Structure. (**A**) A cartoon model depicting the central channel of an E1 helicase domain from a down-the-barrel point of view. The outer circles represent helicase subunits while the structures numbered 1–6 designate the beta hairpin loops. These loops overlap to create a “staircase” pattern. The foot of the beta hairpin staircase is numbered 1. The increasing numbers correspond to higher steps in the staircase. Hairpin loop 2 sits higher than hairpin 1, while 3 overlaps 2, 4 overlaps 3, and so on in an ascending pattern characteristic of E1 hairpins. The histidine residues employed in the untwisting mechanism of melting are denoted in purple at the tip of each hairpin; (**B**) A cartoon model depicting the central channel of an LTag hexameric complex. The six circular domains signify the six helicase subunits while the six oval structures represent beta hairpin loops. The hairpin loops are organized into a planar arrangement characteristic of LTag helicase domains, a distinct organizational method not found in E1 that may contribute to unique melting mechanisms. Histidine residues at the tip of each hairpin are marked in purple [27].

The SV40 core origin for DNA replication (core ori) is composed of four pentanucleotide GAGGC sequences, an AT-rich region (AT), and an early palindromic sequence (EP). From 5’-3’ the ori is composed of the EP, the four pentanucleotides, and the AT region (Figure 5B). Due to the double hexameric nature of LTag, and the asymmetry of the core ori, each hexamer is bound to two GAGGC sequences and either an EP or AT. Once each hexamer is bound, the double hexamer is complete, and completion of the double hexamer is associated with ori melting [23]. The GAGGC sequences themselves are recognized by the OBDs of LTag at major grooves [24,25], while AAA+ regions were found to utilize histidine residues at the tips of beta hairpin loops to interact with ori DNA electrostatically at minor grooves (Figure 6B) [22]. Because proteins often use arginine residues to orient themselves into narrow minor grooves of DNA [26], histidine’s role in the AAA+ domains of LTag was originally thought to be the same as that of arginine elsewhere (i.e., as a DNA recognition element) [22]. However, research into the role of these histidines and their respective beta hairpins has suggested unique models for melting discussed below.

## 7. Mechanisms of Melting with LTag

**Figure 7:**
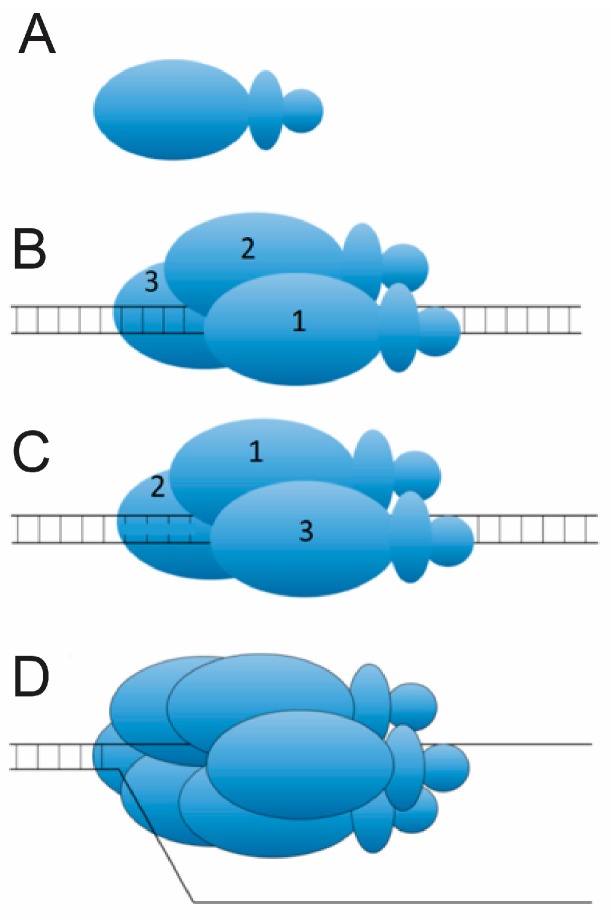
The E1 “Unwinding” Mechanism of Origin Melting. (**A**) An illustration of an E1 monomer. Twelve of these constitute an E1 double hexameric complex shown to unwind DNA after initial melting; (**B**) Pre-twist: Assembly of a single trimer of E1 monomers around dsDNA, and the insertion of histidine residues into the dsDNA; The numbers 1, 2, and 3 demark the three subunits of the trimer. (**C**) Post-twist: The slight rotation, or “twist”, has resulted in a melted origin and reorientation of the three subunits as a result; (**D**) Assembly of a single hexamer of E1 onto ssDNA post melting.

The identified histidine residue is a component of beta hairpin loops which the AAA+ domain utilizes to interact with DNA (Figure 6B). Because LTag is a double hexamer, the dodecahedric complex contains two AAA+ domains with a total of twelve beta hairpin loops, and therefore twelve interactive histidines [28]. Only a single pair of histidines, one imidazole ring from each AAA+ domain, were found to lie in the same minor groove, as well as in the same plane, and within 2.7 angstroms of each other, suggesting the presence of hydrogen bonds to provide enhanced stabilization of the LTag dimer [22]. Portions of the core ori, at which these histidine anchors were found, have been confirmed to be melted after double hexamer assembly [29]. Further mutagenesis of these beta hairpin structures has confirmed their necessity during melting of regions flanking the central pentameric sequences of the core ori [30]. Each set of six beta hairpins are arranged in a planar pattern ultimately creating a ring with a central, positively charged channel (Figure 6B) [31]. This channel is between 7–15 angstroms in diameter [32], making it incredibly unlikely for dsDNA to be thread through, but highly likely for ssDNA [30,33]. For comparison, a hexameric helicase that has been shown to envelop dsDNA, known as RuvB, has a central channel diameter of 30 angstroms [34]. The SV40 distant homolog, E1, utilizes helicase domains determined to envelop solely ssDNA (Figure 7D), and it contains a central channel 17 angstroms in diameter as a result [35]. It is therefore likely that after initial melting, the ssDNA will become engulfed in the central channel as the helicase domains translocate down the DNA, separating the double helix via steric exclusion principles. The steric exclusion model of strand separation occurs when one ssDNA strand, from the duplex that was melted, is enclosed by a hexameric helicase channel so that when the other strand remains outside of the channel, the duplex may be pried apart further by helicase progression down the ssDNA [36]. 

Crystal structures of LTag-DNA complexes have elucidated that each of the OBDs of the double hexamer are oriented 180 degrees to each other when bound to DNA, potentially as a result of a twisting motion which could have generated mechanical force to melt the ori DNA [22]. Since hairpin histidines act as the anchor for LTag’s AAA+ domains, and the minor grooves in which they anchor were subsequently melted, it is feasible that this twisting motion would provide enough force to disrupt hydrogen bonds between base pairs of ori nucleotides, similar to the “untwisting” mechanism utilized by the LTag homolog, E1 (Figure 7). However, LTag-ori-DNA crystal structures showed no significant deformations of DNA [22]. Because of proposals of E1 utilizing trimers in the “untwisting” mechanism before construction of the E1 double hexamers homologous to LTag (Figure 7) [37], it has been proposed that an intermediate LTag structure is formed as well, which melts the ori before the final LTag double hexamer is assembled for translocation [22].

## 8. Structure of the E1 Double Hexamer and Double Trimer 

Much like SV40’s LTag, papillomavirus’s E1 is a homohexameric protein complex responsible for both the initiation of melting and the successive unwinding of DNA. E1 recognizes its unique origin of replication (ori) through DBDs which work to recognize four E1 binding sites, in a nature homologous to SV40’s use of OBDs to bind four GAGGC sequences. From left to right, the E1 protein complex consists of an N-terminal domain, a DBD, an oligomerization domain, a helicase domain, and an acidic C-terminal tail [37]. The DBD is oriented between the two helicase domains which are arranged facing each other. The DBD binds to the E1 binding sites at the center of the ori, while the neighboring helicase domains bind to flanking regions of DNA. The helicase domains of the double hexamer (DH) arrange their beta hairpins in a staircase manner as opposed to the planar formation characteristic of LTag helicase domains (Figure 6).

Unique to studies of E1, formation of an E1 double trimer (DT) has been identified before formation of a double hexamer. Although the exact structure of the DT has not been identified, it is accepted that the DBD of the trimer is oriented between helicase domains, and that the DBD binds the center of the origin while the helicase domains remain bound to flanking regions of DNA. The DT arises when E1 interacts with a dsDNA ori probe in the presence of nucleotides, while the DH subsequently forms in the presence of ATP [37]. The DT has been shown to recognize the origin of replication, and ultimately convert into a double hexamer on ssDNA derived from a melted origin [37].

## 9. Mechanisms of Melting with E1

Although a single E1 trimer does not maintain helicase abilities [38], the DT has conclusively demonstrated an ability to melt dsDNA into ssDNA so that the resulting ssDNA may be used as a template for DH assembly [37]. The identity of the melting complex as DT and not DH, or an intermediate between the two, was concluded through time-course experimentation [39]. The determination of the DT as the melting machinery of E1 has led to recent extensive kinetic and biochemical analyses with the goal of identifying the DT melting mechanism. Plasmid untwisting assays have supported the hypothesis that initial melting is performed via an untwisting mechanism of ori DNA by the DT (Figure 7) [39]. It is proposed that by hydrolyzing ATP, the DT manages to utilize Histidine residues (H507) in the beta hairpins of the helicase domains to initiate melting (Figure 6A). Because the helicase domains themselves remain on the flanks of the E1 binding sites, the histidine interactions with DNA are thought to melt the central portion indirectly through structural deformations of the flanks which propagate through the center binding sites via an untwisting mechanism [39]. If this mechanism were to occur, then mechanical force must be transmitted from the flanks of the ori through the central binding sites. Therefore, nicks in the DNA should inhibit ori melting as a result of interrupting the path of force transference. This is precisely what Shuck and Stenlund found during nicking experiments of ori DNA [39].

The untwisting mechanism has become a widely accepted proposal. However, a “squeeze-to-open” model has been suggested, in which dsDNA is enveloped by DH and ultimately compressed in the central channel of the helicase domains until base pairs are separated [40]. The “squeeze-to-open” model is supported by evidence of melting occurring simultaneously as LTag assembly occurs [29]. Since E1 has only demonstrated central channels capable of enveloping ssDNA, a model involving a larger central channel proves more promising for LTag structures, because they have been shown to undergo conformational changes promoting slight dilations of their central channels [32].

## 10. MCM2-7 Helicase

In eukaryotic cells, it has not yet been determined what melts replication origin DNA. The MCM2–7 helicase [41] and the origin recognition complex (ORC) [42] assemblies are the most likely candidates, since these complexes hydrolyze ATP, and energy is required for origin melting. The MCM2–7 helicase is related to Large T and E1 helicase proteins, suggesting conservation of mechanism [43]. However, MCM2–7 lacks much of the machinery present in the viral counterparts, suggesting that the mechanism for origin melting is different for MCM2–7 compared to Large T and E1 [43]. Furthermore, the MCM2–7 helicase is very weak on its own [44], and MCM2–7 requires Cdc45 and GINS attachment for full helicase activity [45]. The CMG (CDC45-MCM2–7-GINS) helicase is conserved in archaea as well [46].

The MCM2–7 has an N-terminal domain, required for double hexamer attachment, and a C-terminal AAA+ domain, required for ATPase activity [41,47]. The double-hexamer interface is active during late M and G_1_ phase, when the MCM2–7 is loaded as a double hexamer [47,48]. However, during S phase, when the replication fork helicase is activated, the MCM2–7 double hexamers dissociate, and the resulting CMG helicases unwind bidirectionally from the origin [49,50]. The MCM2–7 helicase also has DNA binding regions within the N-terminal and AAA+ domains [51,52]. It is generally agreed that the CDC45-MCM2–7-GINS assembly, the fully-active helicase, unwinds DNA by a steric exclusion mechanism [49,53,54,55]. In this model, the leading strand passes through the central channel of CMG [49,53,54,55]. The excluded lagging strand may pass through a side channel of the CMG, or alternatively the lagging strand may pass completely outside the CMG [49,53,54,55]. In either event, the double-stranded origin DNA must be melted to activate CMG unwinding.

What is the mechanism for replication fork unwinding by the CMG? According to the rotary model, the ssDNA lying inside the central channel of CMG is passed from one AAA+ domain to another in a sequential manner [35]. This model is derived mainly from homology to the Large T and E1 viral helicase systems, for which a rotary model is proposed [35]. A second model, based upon recent electron microscopy structures, proposes that the ssDNA binding regions of the AAA+ domain hands-off the ssDNA to the ssDNA binding region within the N-terminal region [49,53,54,55]. Future studies may reveal which one of these two models reflects the CMG mechanism for unwinding DNA in vivo.

The origin dsDNA encircled by MCM2–7 must be converted from dsDNA to ssDNA during replication initiation. In budding yeast, the origins are AT-rich, similar to the origins of bacteria and eukaryotic viral origins, suggesting that this may be conserved to promote initial melting of the origin, since AT-rich regions are inherently prone to melting. The MCM2–7 may open to promote exclusion of the lagging strand during the replication initiation. However, the mechanism for MCM2–7 ring opening is currently not known, but it may occur at the MCM2–MCM5 interface because this interaction surface is inherently weak [44,56,57]. Future studies may reveal how the MCM2–7 ring opens during S phase to allow for origin melting, and future studies may also reveal whether ring opening occurs before or after MCM2–7 double hexamer dissociation.

Additional ssDNA binding proteins may participate in the origin melting process. Proteins that bind origin ssDNA in budding yeast include MCM10 [58,59], SLD3 [60], SLD2 [61], DPB11 [62], and RPA [63], the eukaryotic single-stranded binding protein. These proteins do not hydrolyze ATP, and therefore their contribution to origin melting lies in their ability to bind ssDNA and stabilize the melted state. Interestingly, mutating the ssDNA binding residues of MCM10, SLD2, SLD3, and DPB11 results in decreased replication initiation and diminished recruitment of RPA to replication origins [62,64,65,66]. These data suggest that one or more of these initiation factors may be required to stabilize melted origin ssDNA, and perhaps even hand off melted origin DNA to RPA. However, little is known regarding the mechanism for how the initiation factors melt origin DNA, and little is known how the initiation factors hand off ssDNA to RPA. The human homologs of MCM10 (human MCM10) [67,68], SLD3 (Treslin) [66], and SLD2, RECQL4 [69], have also been shown to bind ssDNA, suggesting that the function may be conserved from budding yeast to human. 

A replication initiation assay has recently been reconstituted for budding yeast using only purified proteins [70]. Furthermore, methods exist in budding yeast for the induced-degradation of essential genes, with phenotypic scoring of the mutant phenotype [71,72]. In addition, the ssDNA binding residues of the initiation factors have now been identified for budding yeast [62,64,65,66]. Thus, through a combination of in vitro reconstitution assays and in vivo experiments, a mechanistic understanding of how origin DNA is melted, stabilized, and transferred to RPA will soon be revealed for this model eukaryotic organism.

## 11. Concluding Remarks

A key step in replication initiation in all organisms may be the melting of origin DNA, since replication fork helicases in all systems seem to unwind DNA by a steric exclusion mechanism. In bacteria, the DnaA protein may be responsible for melting origin DNA, and also for loading the helicase onto the melted ssDNA. For eukaryotic viruses, the Large T and E1 helicases are competent to melt the origin DNA and subsequently unwind the DNA by steric exclusion. For the cellular eukaryotic replication initiation machinery, it appears that essential initiation factors, including MCM10, SLD3, SLD2, and DPB11, may be responsible for stabilizing the melted origin DNA, and these proteins may also participate in the hand-off of melted origin ssDNA to RPA. Thus, while origin melting is common for all domains of life, the mechanism for origin melting may be quite different for each DNA replication initiation system.
